# Flow cytometric detection of hyper-polarized mitochondria in regulated and accidental cell death processes

**DOI:** 10.1007/s10495-020-01613-5

**Published:** 2020-06-03

**Authors:** G. Warnes

**Affiliations:** Flow Cytometry Core Facility, The Blizard Institute, Barts and The London School of Medicine and Dentistry, Queen Mary London University, 4 Newark Street, London, E1 2AT UK

**Keywords:** Mitochondrial function, ROS, RCD, ACD, Parthanatos, DNA damage

## Abstract

Shikonin induced necroptosis in Jurkat cells were identified flow cytometrically by the up-regulation of RIP3 in live cells and that a proportion of these cells underwent other forms of regulated cell death (RCD) which included parthanatos (< 10%), or cleaved PARP (< 10%) and DNA Damage (> 30%). Live necroptotic cells also possessed functioning mitochondria with hyper-polarized mitochondria membrane potential and generated a fivefold increase in cellular reactive oxygen species (ROS) which was resistant to inhibition by zVAD and necrostatin-1 (Nec-1). After loss of plasma membrane integrity these dead necroptotic cells then showed a higher incidence of parthanatos (> 40%), or cleaved PARP (> 15%) but less DNA Damage (< 15%). Inhibition of shikonin induced apoptosis and necroptosis by zVAD and Nec-1 respectively resulted in live necroptotic cells with an increased incidence of cleaved PARP and reduced levels of DNA Damage respectively. Dead necroptotic cells then showed a reduced incidence of parthanatos and DNA Damage after inhibition by zVAD and Nec-1 respectively. A high proportion of these dead necroptotic cells (30%) which lacked plasma membrane integrity also displayed functioning hyper-polarized mitochondria with high levels of cellular ROS and thus had the capacity to influence the outcome of RCD processes rather than just been the end product of cell death, the necrotic cell. Flow cytometry can thus measure multiple forms of RCD and the level of cellular ROS and MMP which highlights the inter-connection between cell death processes and that a single cell may simultaneously display multiple forms of RCD.

**Electronic supplementary material:**

The online version of this article (10.1007/s10495-020-01613-5) contains supplementary material, which is available to authorized users.

## Introduction

**Fig. 1:**
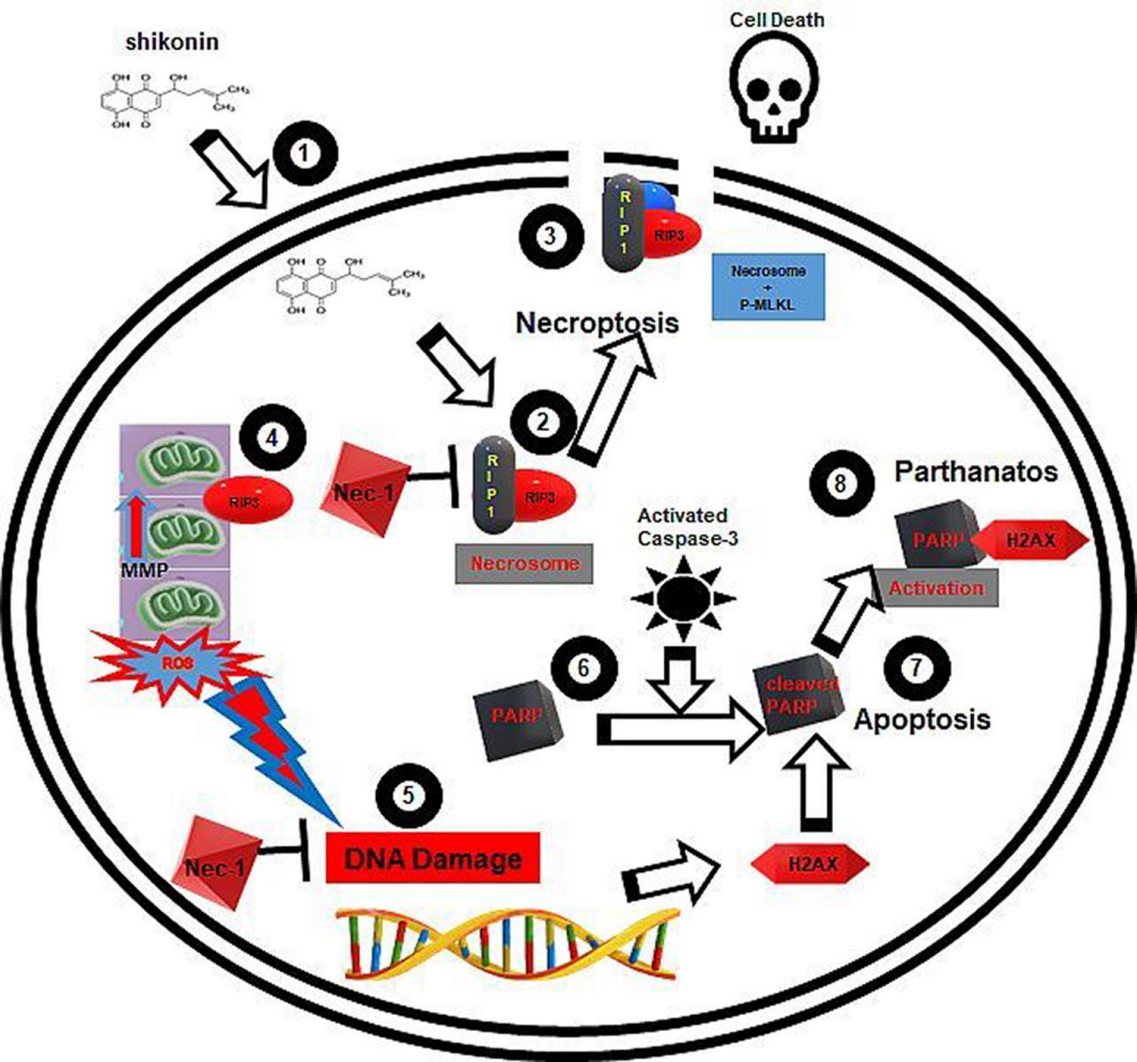
Shikonin induction of necroptosis, apoptosis, DNA Damage, cleaved PARP and parthanatos. (1) Shikonin is taken up by the cell which causes (2) RIP1 auto-phosphorylates which phosphorylates RIP3 to form the necrosome which can be blocked by Nec-1. (3) The necrosome then phosphorylates MLKL leading to the permeablisation of the plasma membrane and cell death by necroptosis. (4) Phosphorylated RIP proteins move to the mitochondria leading to raised MMP or hyperpolarization of mitochondria and excessive production of ROS. (5) ROS causes DNA Damage leading to phosphorylation of histone γH2AX which can be blocked by Nec-1. (6) PARP is cleaved by active caspase-3 which is generated during apoptosis (7). Parthanatos occurs when phosphorylated γH2AX hyper-activates cleaved PARP (8)

The regulated necrotic cell death process (RCD) necroptosis is a caspase independent process which after TNFα binding to the TNF receptor becomes associated with TRADD (TNF receptor type 1-associated with death domain protein) and TRAF-2 (TNFR-associated factor 2 forming complex I [1–6]). This complex together with the RIP1 protein then auto-phosphorylates after inhibition of caspase-8 (active caspase-8 cleaves RIP1), [7] which then acts as the first ROS sensor in this form of regulated cell death [2–6, 8]. This ultimately leads to the up-regulation and phosphorylation of RIP3 forming the so called necrosome and to the recruitment and phosphorylation of MLKL (pseudokinase mixed lineage kinase domain-like protein) at the plasma membrane leading to cell lysis Fig. 1 [5, 6, 9–12]. RIP3 has also been reported to move to the inner mitochondrial membrane where it’s interaction with PDC, CamK II, Drp1 and PGAM5 leads to the enhanced production of ROS by mitochondria Fig. 1 [3, 13, 14]. ROS generated during necroptosis has been previously shown to enhance auto-phosphorylation of RIP1 and was essential for RIP3 recruitment to the necrosome leading to enhanced formation of functional necrosomes, with Reactive Oxygen Species (ROS) acting in a positive feedback loop ensuring maintenance of necroptosis (Fig. 1). RIP1 is mainly located in the cytoplasm in the form of the necrosome as part of the necroptosis process, however nuclear RIP1 has also been shown to associate in a kinase independent manner and activate PARP1 inducing PARP1-dependent regulated necrosis or parthanatos Fig. 1 [15].

The drug shikonin is a naturally occurring naphthoquinone has like TNFα [4, 16] been reported to induce apoptosis and necroptosis, as well as generate ROS (Fig. 1 [3, 8, 17, 18]. Necrostatin-1 (Nec-1) has been reported to inhibit necroptosis and also the generation of ROS by mitochondria but to a lesser extent in the cytoplasm Fig. 1 [3, 8, 16]. Shikonin has also been reported to increase the level of γH2AX or DNA Damage (as part of the DNA Damage Response, DDR), cleaved PARP and also the hyper-activation of cleaved PARP which zVAD can down-regulate Fig. 1 [8, 19]. The action of cleaved PARP is also known to reduce levels of NAD + resulting in lower ATP levels which has been reported to act as a molecular switch towards programmed necrosis [15]. RIP1 is not only located in the cytoplasm which together with RIP3 forms the necrosome, but parthanatos is also found in the nucleus associated with activated PARP1 and RIP1 in the development of a parthanatos response [19–21]. RIP3 has also been reported to regulate levels of mitochondrial ROS in glioma cells which in turn increased the incidence of double strand breaks in DNA (DNA DSBs) leading to an increase in γH2AX or DNA Damage resulting in chromatinolysis and necroptosis [8]. Jurkat cells have previously been shown to display no change in the high level of DNA Damage in live necroptotic cells (reduced in dead cells) after treatment with shikonin [18]. Shikonin treatment of Jurkat cells were also reported to show increased hyper-activation of PARP by γH2AX and cleaved PARP in live populations as well as dead necroptotic and oncotic cells (RIP3^−ve^/Caspase-3^−ve^) [15, 16, 18]. Thus live necroptotic cells are composed of different sub-populations of cells undergoing caspase-3 independent cleaved PARP, parthanatos, DNA Damage or just RIP3 up-regulated necroptosis [15, 16, 18].

Mitochondrial activity during the necroptotic process has been misreported by the misuse of mitochondrial functional probes without using cell viability probes when performing flow cytometry [14, 22]. The non-exclusion of dead cells induced by the cytotoxic drug in question leads to a massive under-reporting of the degree of MMP in the remaining live cells. This study investigates this discrepancy by using a cell viability probe to show changes in MMP and ROS generation in live cells undergoing necroptosis as well as oncosis, early, late and RIP1-dependent apoptosis and relates this to parthanatos, cleaved PARP expression and the degree of DNA Damage during RCD and ACD (Accidental Cell Death) processes. Further studies investigating the modulation of such responses to shikonin by blockade of necroptosis or apoptosis by Nec-1 or zVAD were also performed. This flow cytometric approach in the study of multiple forms of RCD and ACD highlights the presence of functional hyper-polarized mitochondria with high levels of ROS in live and apparently dead necroptotic cell populations.

## Materials and methods

### Induction of necroptosis and apoptosis

Jurkat cells (human acute T cell leukaemia cell line) were grown in RPMI 1640 medium with 10% FBS and penicillin/streptomycin (Invitrogen, UK) at 37 °C and 5% CO_2_ and either left untreated or treated with 0.5 μM Shikonin (Santa Cruz, USA) for 24 h. Cells were also pre-treated with the pan-caspase blocker zVAD (20 μM, Enzo Life Sciences, USA) or the necroptosis blocker necrostatin-1 (60 μM, Cambridge Bioscience, UK) for 2 h before and during the treatment with 0.5 μM Shikonin for 24 h.

### Live cell labelling

After treatment part of the harvested cells (1 × 10^6^) were labelled with Violet Live Cell Caspase (2 μl/ml, Becton Dickinson, USA) and 500 nM CellROX Green (Invitrogen, USA) at 37 °C for 1 h. Then incubated with 50 nM MitoTracker Deep Red (Invitrogen, USA) and fixable live dead stain Zombie NIR (Near Infra-Red) (BioLegend, UK) at 37 °C for 15 min. 100,000 events were analysed on an ACEA Bioscience Novocyte 3000 flow cytometer.

### Intracellular labelling protocol

The remaining Jurkat cells were labelled with fixable live dead stain Zombie NIR for 15 min at RT and washed. Pelleted cells were sequentially fixed in Solution A (CalTag, UK) then permeabilised with 0.25% Triton X-100 (Sigma, UK) for 15 min each at RT. Jurkat cells (2 × 10^6^) were incubated for 20 min at RT with 2 μl of anti-RIP3-PE (clone B-2, Cat. No. sc-374639, Santa Cruz, USA), cleaved anti-PARP-PE-CF594 (clone F21-852, Becton Dickinson, USA), anti- γH2AX -PE-Cy7 (clone 2F3, BioLegend, UK) and anti-active caspase-3-BV650 (clone C92-605, Becton Dickinson, USA) for 20 min at RT. Washed cells were resuspended in 400 μl PBS and analysed on a ACEA Bioscience Novocyte 3000 flow cytometer and 200,000 events analysed.

### Flow cytometry

MitoTracker Deep Red and Zombie NIR were excited by the 633 nm laser and collected at 660/20 nm and 780/60 nm. Caspase Violet and Caspase-3-BV650 were excited by the 405 nm laser and collected at 450/20 nm and 675/30 nm. CellROX Green, RIP3-PE, cleaved PARP-PE-CF594, γH2AX -PE-Cy7 were excited by the 488 nm laser and collected at 530/20 nm, 572/28, 615/20, 780/60 nm respectively. Single colour controls were employed to determine the colour compensation using the pre-set voltages on the instrument using Novo Express software (ver 1.2.5, ACEA Biosciences, USA).

### Gating strategies

**Fig. 2:**
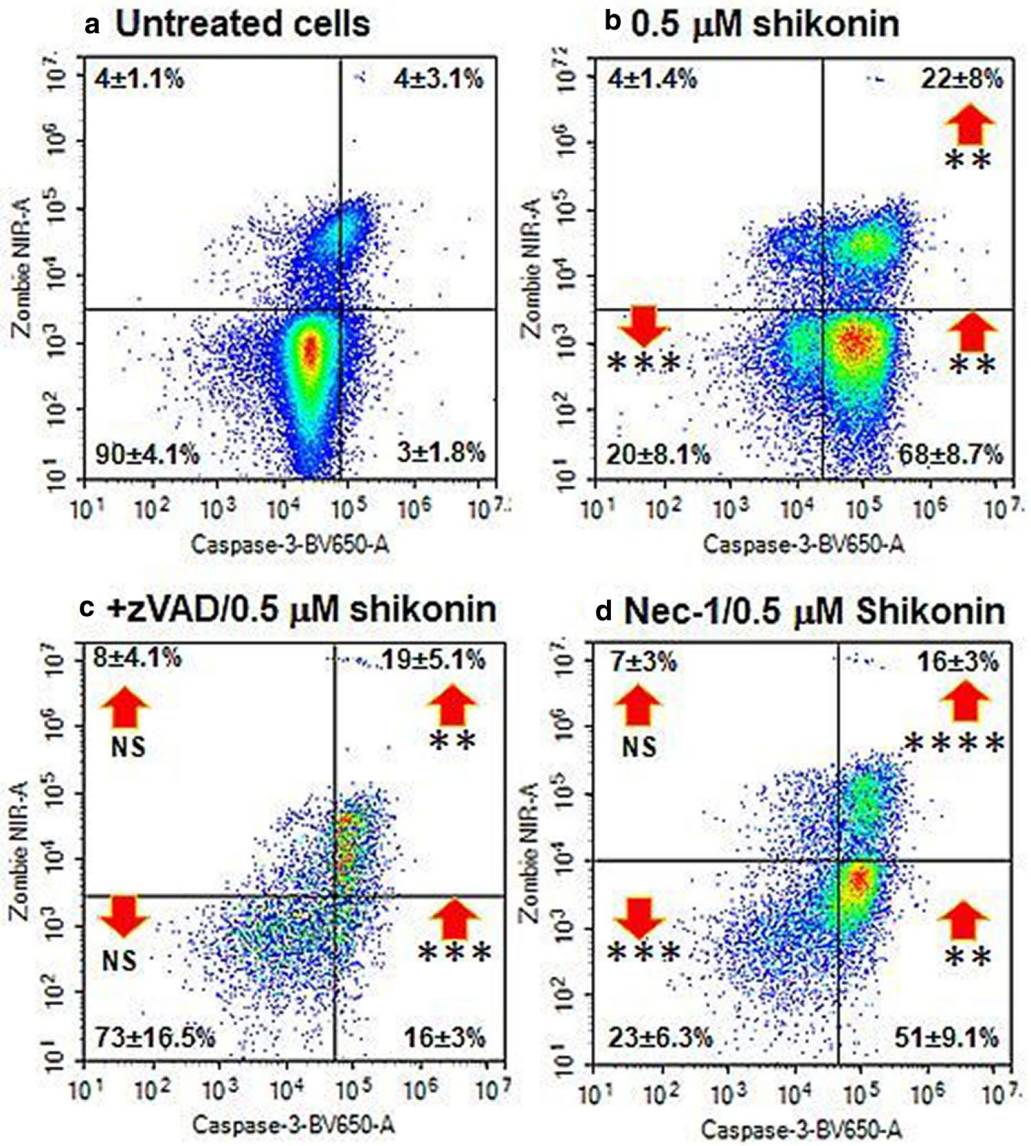
Cell death and caspase-3 activation assay. Jurkat cells were untreated (**a**), treated with 0.5 μM shikonin for 24 h (**b**), pre-treated with 20 μM zVAD for 2 h then with 0.5 μM shikonin for 24 h (**c**), pre-treated with 60 μM necrostatin-1 (Nec-1) for 2 h then with 0.5 μM shikonin for 24 h (**d**). Live (DN), early apoptotic (EAPO, Zombie NIR^−ve^/caspase 3^+ve^), late apoptotic (LAPO, Zombie NIR^+ve^/caspase 3^+ve^) and oncotic cells (Zombie NIR^+ve^/caspase 3^−ve^). Average % ± SD (n = 3) were analysed for significance by One Way ANOVA (*P* < 0.5) and post ad hoc tested for significance, NS (*P* > 0.5, not significant), significance was *P* < 0.05*, *P* < 0.01**, *P* < 0.001***, *P* < 0.0001****, with red arrows indicating change compared to untreated cells (Color figure online)

Unfixed cells were gated on FSC vs SSC removing the small debris near the origin with single cells gated on an FSC-A vs FSC-H dot-plot. This was followed by a dot-plot of Caspase-Violet vs Zombie NIR with a quadrant placed marking off live cells in the double negative quadrant (lower left), early apoptotic cells were Caspase Violet^+ve^/Zombie NIR^−ve^ (lower right) and lastly with dead cells labelled with Caspase Violet^+ve^ or ^−ve^/Zombie NIR^+ve^ (upper quadrants) indicating late apoptotic and necrotic/oncotic cells respectively (Fig. 1S). Live, early and late apoptotic and oncotic populations were gated for CellROX Green vs MitoTracker Deep Red with upper-left quadrant CellROX Green^−ve^/MitoTracker Deep Red^+ve^ or live cells with mitochondrial function but without ROS. The upper right quadrant being CellROX Green^+ve^/MitoTracker Deep Red^+ve^ or cells with mitochondrial function and ROS, lower right quadrant being CellROX Green^+ve^/MitoTracker Deep Red^−ve^ or cells without mitochondrial function but with ROS (Fig. 2S). MitoTracker Deep Red and CellROX Green signals from shikonin treatments were referenced to untreated cells (100%) then expressed as percentage fold increase, see Fig. 5.

**Fig. 3:**
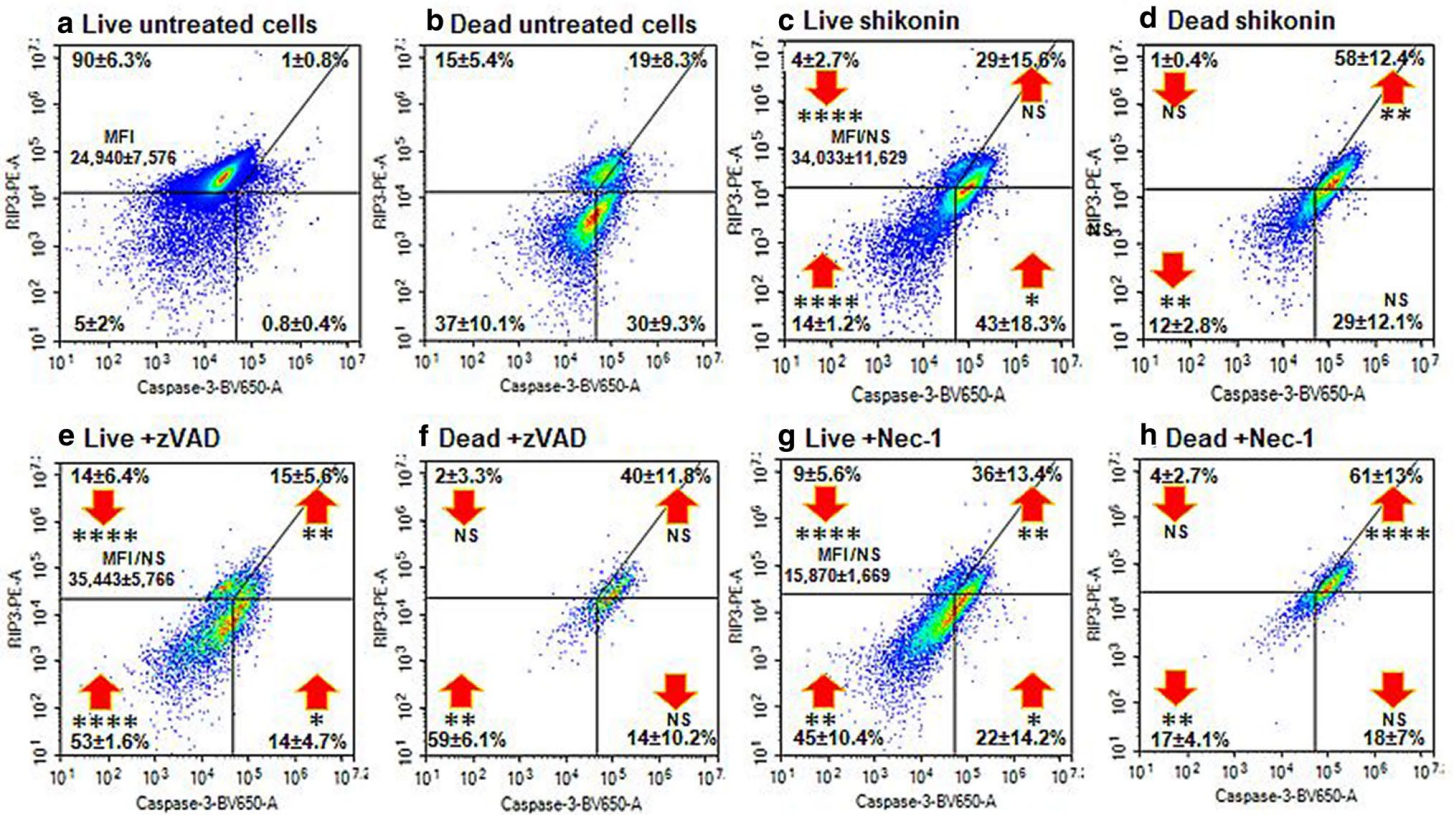
RIP3 and caspase-3 activation assay. After gating on live and dead cells from a Zombie NIR vs. Caspase-3-BV650 dot-plot cells were then analysed on a RIP3-PE vs. Caspase-3-BV650 dot-plot. The necroptotic phenotype indicated by RIP3^+ve^/^high+ve^/Caspase-3^−ve^ which has up-regulated RIP3 Median Fluorescent Intensity (MFI). The apoptotic phenotype was RIP3^−ve^/Caspase-3^+ve^, RIP1-dependent apoptosis (RIP3^+ve^/Caspase-3^+ve^) and quadruple negative (RIP3^−ve^/Caspase-3^−ve^). Live (**a**) and dead (**b**) untreated cells, or cells treated with 0.5 μM shikonin for 24 h (**c**, **d**), pre-treated with 20 μM zVAD for 2 h then with 0.5 μM shikonin for 24 h (**e**, **f**) or pre-treated with 60 μM Nec-1 for 2 h then with 0.5 μM shikonin for 24 h (**g**, **h**). Average Median Fluorescent Intensity MFI or average % ± SD (n = 3) were analysed for significance by One Way ANOVA (*P* < 0.5) and post ad hoc tested for significance, NS (*P* > 0.5, not significant), significance was *P* < 0.05*, *P* < 0.01**, *P* < 0.001***, *P* < 0.0001****, with red arrows indicating change compared to untreated cells (Color figure online)

After Zombie NIR labelling, fixation, permeabilisation and intracellular labelling with antibodies (listed in the "Intracellular labelling protocol" section). Cells were then analysed by gating on a dot-plot of Caspase-3-BV650 vs Zombie NIR with a quadrant placed marking off live cells in the double negative quadrant (lower left), with Caspase-3-BV650^+ve^/Zombie NIR^−ve^ (lower right) indicating early apoptotic (EAPO) cells (Fig. 3S, a). Lastly, Caspase-3-BV650^+ve or −ve^/Zombie NIR^+ve^ upper quadrants indicated late apoptotic (LAPO) and oncotic cells, respectively (Fig. 3S, a). Live (including early apoptotic) and both dead cell populations were gated separately and analysed in RIP3 vs Caspase-3 dot-plots with RIP3^+ve^/Caspase-3^−ve^ which indicated live cells or necroptosis when RIP3 Median Fluorescence Intensity (MFI) was up-regulated (Fig. 3S, b, d). RIP3^−ve^/Caspase-3^+ve^ cells indicate those that had undergone early (EAPO) or late apoptosis (LAPO, Fig. 3S, b, d). Double positive events indicate cells of the RIP1-dependent apoptosis phenotype (RIP1APO, Fig. 3S, b, d). Double negative (DN) cells and all other phenotypes were further analysed for γH2AX and cleaved PARP expression (Fig. 3S, c, e). The following populations were identified: DNA Damage (DDR) phenotype were γH2AX^+ve^/PARP^−ve^ events, γH2AX hyper-activation of cleaved PARP (Hyper-Act) or in the absence of caspase-3, parthanatos γH2AX ^+ve^/PARP^+ve^, cleaved PARP (PARP) as γH2AX ^−ve^/PARP^+ve^ and negative (QN, γH2AX ^−ve^/PARP^−ve^) as shown in Fig. 3S, c, e.

### Statistics

All experiments (at least n = 3) were expressed either as average percent positive ± SD, average Median Fluorescence Intensity (MFI) ± SD and percent average Median Fluorescence Intensity (MFI) normalised to untreated cells ± SD. Data were tested for multivariate normality with the Kolmogorov–Smirnov goodness of fit test, followed by One Way ANOVA (*P* < 0.5) and then post ad hoc to test for significance (*P* < 0.5) between treatments with GraphPad PRISM ver. 8.4.0 software Inc., USA. Not considered significant *P* > 0.05 (NS), significance was either *P* < 0.05*, *P* < 0.01**, *P* < 0.001***, *P* < 0.0001**** when treated cells were compared to untreated and also between treatments when analysing γH2AX and PARP.

## Results

### Induction of necroptosis and apoptosis

**Fig.4:**
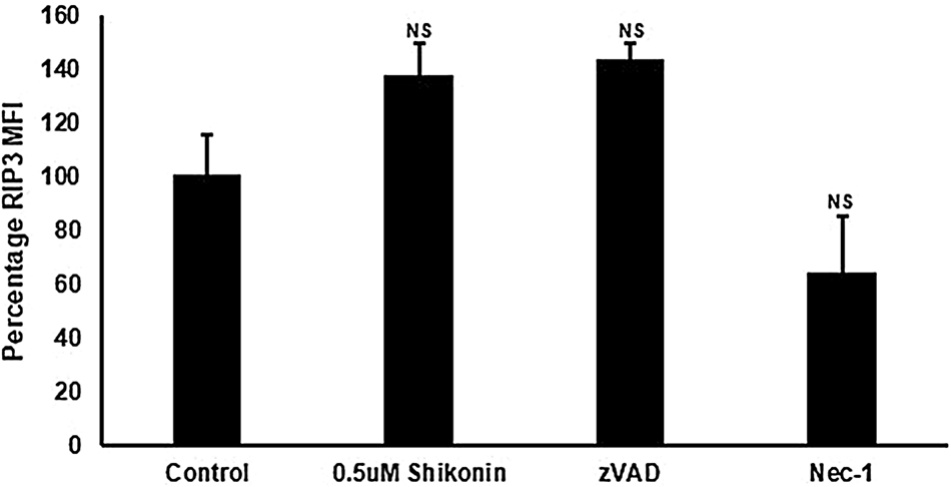
Analysis of RIP3 expression. Live cells expressing RIP3 but not caspase-3 were analysed for RIP3 expression for untreated cells, then average percentage in RIP3 MFI ± SD were determined for 0.5 μM shikonin (24 h), 0.5 μM shikonin with 20 μM zVAD or 0.5 μM shikonin with 60 μM Nec-1. Average percentage of RIP3 MFI ± SD (n = 3), were analysed for significance by One Way ANOVA (*P* < 0.5) and post ad hoc tested for significance, NS (*P* =  > 0.5, not significant), significance was *P* < 0.05*, *P* < 0.01**, *P* < 0.001***, *P* < 0.0001**** compared to untreated cells

Jurkat cells treated with shikonin showed a significant increase in early (68%) and late apoptosis (22%, Fig. 2a, b). Shikonin induced necroptosis (RIP3^high+ve^/Caspase-3^−ve^) with an up-regulation of RIP3 (37%) and RIP1-dependent apoptosis (29%, RIP3^+ve^/Caspase-3^+ve^) compared to untreated live cells (Figs. 3a, c and  4). The dead cells showed increased levels of RIP1-dependent apoptosis compared to untreated cells (58%, Fig. 3b, d).

**Fig. 5:**
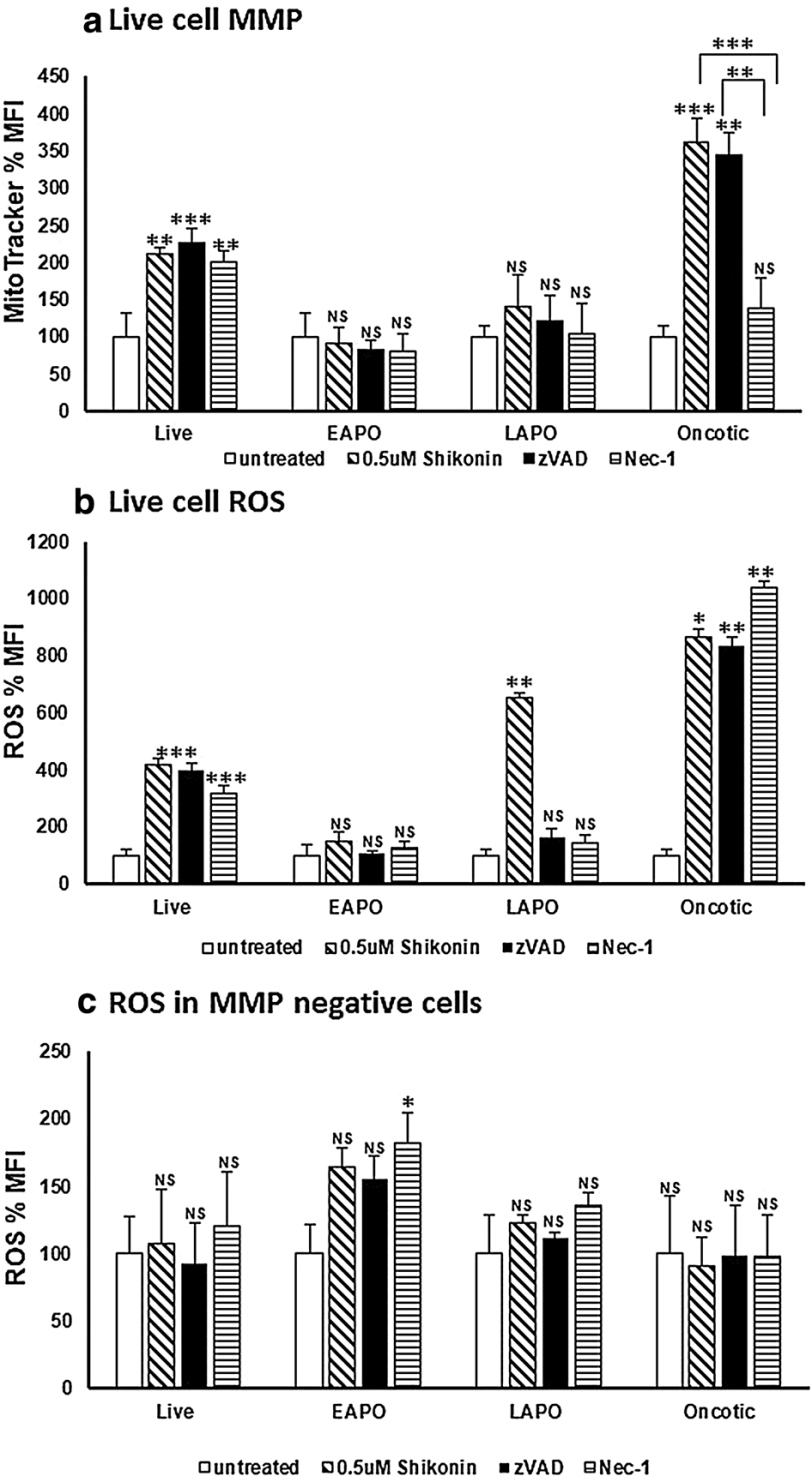
Mitochondrial MMP, function and cellular ROS analysis. Jurkat cells were loaded with violet caspase, Zombie NIR, MitoTracker Deep Red and CellROX Green as described in the Materials and Methods section. Live (DN), early apoptotic (EAPO, Zombie NIR^+ve^/caspase violet^+ve^), late apoptotic (LAPO, Zombie NIR^+ve^/caspase violet^+ve^) and oncotic (Zombie NIR^+ve^/caspase violet^−ve^) cells were analysed for MMP by MitoTracker Deep Red (**a**), those MMP + ve cells analysed for CellROX Green, ROS (**b**), those MMP-ve cells analysed for CellROX Green, ROS (**c**). Untreated cells, 0.5 μM shikonin, blockade of shikonin with zVAD (20 μM) or blockade of shikonin with Nec-1 (60 μM) were analysed. Mean percentage of MFI was normalised to untreated cells ± SD, (n = 3) were analysed for significance by One Way ANOVA (*P* < 0.5) and post ad hoc tested for significance, NS (*P* > 0.5, not significant), significance was *P* < 0.05*, *P* < 0.01**, *P* < 0.001***, *P* < 0.0001**** compared to untreated cells and between treatments as indicated

Treated live cells were comprised of those undergoing necroptosis (4%, RIP3^high+ve^/Caspase-3^−ve^) or were double negative (DN) for RIP3/Caspase-3 (14%) together which showed a significant two and four fold increase in MMP and ROS respectively compared to untreated live cells (Figs. 2S, a, e; 3c and 5a, b). There was also a rise in live cells that lacked mitochondrial function but showed no significant rise in ROS (13% compared to untreated 2%, Figs. 2S, a, e and  5c). Early apoptotic treated cells mainly lacked MMP (while untreated did not) but generated > 60% more ROS (Figs. 2S, b, f and 5c). The remaining treated early apoptotic cells with mitochondrial function (7%) generated more ROS (50%) than untreated early apoptotic cells (Figs. 2S, b, f and 5a, b).

Late apoptotic cells mainly lacked mitochondrial function, but generated 23% more ROS than untreated cells (Figs. 2S, c, g and 5c). However, the small percentage of late apoptotic cells (2%) with raised MMP (40%) with a 6.5-fold increase in ROS (Figs. 2S, c, g and 5a, b). Oncotic cells (Zombie^+ve^/Caspase violet^−ve^) which comprised of dead necroptotic and dead oncotic DN cells showed a high degree of mitochondrial function (30% compared to untreated cells 5%) with raised MMP (3.6 fold) and eightfold–ninefold more ROS than untreated oncotic cells (Figs. 2S, d, h and 5a, b). Those oncotic cells without mitochondrial function showed no increase in detectable ROS (Figs. 2S, d, 5c).

**Fig. 6:**
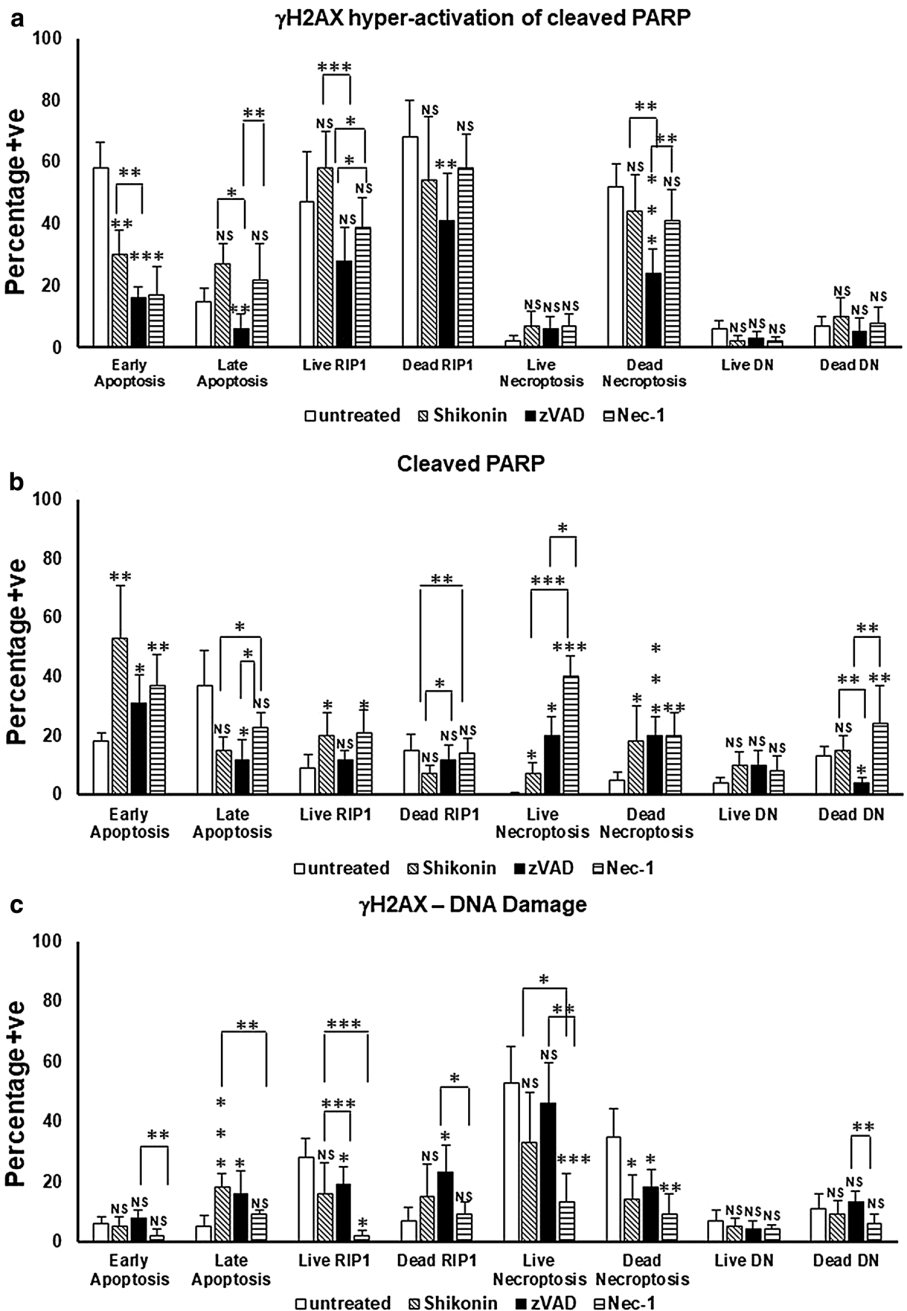
γH2AX hyper-activation of cleaved PARP or parthanatos, cleaved PARP and γH2AX or DNA Damage assay. Jurkat untreated, treated with 0.5 μM shikonin or pre-treated zVAD (20 μM) or Necrostatin-1 (60 μM) for 2 h then incubated with 0.5 μM shikonin for 24 h. After gating on live and dead cells from a Zombie NIR vs. Caspase-3-BV650 dot-plot untreated or treated live and dead Jurkat cells were analysed on a RIP3-PE v Caspase-3-BV650 dot-plot. From which early and late apoptotic, necroptotic, RIP1-dependent apoptotic and double negative (DN) populations were analysed for γH2AX and cleaved PARP, see Figs. 1S and 2S for detailed information. The incidence of γH2AX hyper-activation of cleaved PARP or parthanatos (**a**), cleaved PARP (**b**) and DNA Damage (**c**) were determined for all populations listed above. Average % ± SD, (n = 3) were analysed for significance by One Way ANOVA (*P*  < 0.5) and post ad hoc tested for significance, NS (*P* > 0.5, not significant), significance was *P* < 0.05*, *P* < 0.01**, *P* < 0.001***, *P* < 0.0001**** compared to untreated cells and between treatments as indicated

Live cells showed a small increase in the incidence of γH2AX hyper-activation of cleaved PARP or parthanatos in live necroptotic, RIP1-dependent apoptosis and also late apoptotic cells, with a decrease observed in early apoptotic cells (Fig. 6a). The incidence of cleaved PARP was significantly increased after shikonin treatment in early, live RIP1-dependent apoptosis and both necroptotic populations; with decreases in late apoptosis and dead RIP1-dependent apoptosis (Fig. 6b). DNA Damage was correspondingly decreased after drug treatment in both necroptotic populations and live RIP1-dependent apoptosis, with an increase observed in late apoptotic cells (Fig. 6c).

### zVAD blockade of apoptosis

Blockade of shikonin induced apoptosis by zVAD showed a decrease in early apoptosis (16%, Fig. 2c). The incidence of live necroptotic cells with raised RIP3 MFI was increased (14% compared to 4% treated cells, Figs. 3e and 4). Early/late and RIP1-dependent apoptosis was reduced compared to drug treatment (Figs. 3e, f and 4). Correspondingly there was a significant increase in live and dead oncotic DN populations compared to drug and untreated cells (Fig. 3a–f).

The live cells after zVAD blockade of shikonin comprised of 14% necroptotic and 53% DN compared to 4% and 14% in treated cells respectively (Fig. 3c, e). These live cells showed a doubling of cells without mitochondrial function which generated ROS (Figs. 2S, i and 5c). Consequently, the incidence of double positive live cells was reduced (56%) compared to treated cells (70%) but had the same high level of MMP as that induced by shikonin treatment (two fold increase) and ROS (fourfold increase, Figs. 2S, i and 5a, b). Early and late apoptotic cells after zVAD blockade of shikonin showed an abrogation of ROS generated by cells with functioning mitochondria with no change in the level of ROS in cells without mitochondrial function compared to drug treatment (Figs. 2S, b, c, f, g, j, k and 5). The incidence of apparently live oncotic cells after zVAD blockade was reduced to untreated levels but with the maintenance of the high level of MMP and ROS (Figs. 2S, d, h, l and 5a, b). However, the incidence of cells without mitochondrial function but generated the same level of ROS was increased (15%) compared to drug and untreated cells (< 5%, Figs. 2S, d, h, l and 5c).

γH2AX hyper-activation of cleaved PARP or parthanatos and cleaved PARP was reduced in most cell populations after zVAD blockade of shikonin compared to drug treatment except live necroptotic cells which showed an increase in cleaved PARP (Fig. 6a, b). In contrast the DNA Damage populations was increased in most cell populations after zVAD blockade of shikonin compared to drug treatment (Fig. 6c).

### Necrostatin-1 blockade of necroptosis

Nec-1 blockade of shikonin reduced early apoptosis (51%) compared to drug treatment (68%, Fig. 2d). Such treatment blocked necroptosis as indicated by the abrogation of RIP3 up-regulation (Figs. 3g and 4). While, late apoptosis was reduced, the incidence of the live DN population (45%) was increased compared to drug treatment (14%, Fig. 3c, d, g, h).

This increased the percentage of live DN cells (45%, Fig. 3g) resulted in a reduction in live cells with functioning mitochondria (49% compared to 70% by drug treatment) but which still displayed increased MMP and ROS by 2 and threefold respectively (Figs. 2S, a, e, m and  5a, b). Early apoptotic cells showed no change in the degree of ROS generation in cells with or without mitochondrial function (Figs. 2S, b, c, n, o and 5). While, late apoptotic cells with dysfunctional mitochondria showed no significant rise in generated ROS levels above untreated cells, the few cells with functional mitochondria showed an abrogation of ROS (Fig. 4b, c). Oncotic cells with dysfunctional mitochondria showed no change in ROS after Nec-1 blockade, while those with functioning mitochondria were reduced (< 4%) and showed an abrogation of the raised MMP (> 40%) but had a ten fold increase in ROS compared to untreated cells (Figs. 2S, d, p and 5a, b, c).

The incidence of γH2AX hyper-activated cleaved PARP expressing cells was reduced after Nec-1 blockade of shikonin in only early and live RIP1-dependent apoptotic populations compared with drug treatment, with no change observed in other populations (Fig. 6a). Cleaved PARP was increased by Nec-1 in late apoptotic, live necroptotic and dead oncotic DN cells compared to drug treatment, with a reduction observed in the early apoptotic population (Fig. 6b). In contrast, all cells after Nec-1 blockade showed reductions in DNA Damage as opposed to the increase observed after zVAD blockade of shikonin (Fig. 6c).

## Discussion

The main aim of this flow cytometric based study was to show that shikonin induced Jurkat cell necroptosis and apoptosis (blocked by zVAD and Nec-1) resulted in measurable changes in mitochondrial function, MMP and cellular ROS in live, early, late apoptotic and oncotic cell populations. Unfortunately, the lack of reliable MitoTracker and CELLROX Green data after fixation and permeabilisation has not allowed analysis of MMP and ROS in specific cell death populations (*e.g.* live necroptotic cells undergoing parthanatos). The assay also tracked the incidence of shikonin (blocked by zVAD or Nec-1) induced necroptosis, caspase-3 dependent apoptosis, RIP1-dependent apoptosis, DN populations (live and dead oncotic DN cells) as well as the incidence of parthanatos (or γH2AX hyper-activation of PARP), cleaved PARP and DNA Damage in these populations. Other studies imply that the necroptosis process is typified by the presence of dysfunctional mitochondria and high levels of ROS, this was mainly due to the misreporting of MitoTracker data due to the lack of a cell viability probe [14, 22]. Cytotoxic drugs usually cause a high degree of cell death with the possibility that the remaining live cells (with functioning mitochondria) are thus hidden by the dead cell population (without functioning mitochondria) leading to a misreporting of the health of mitochondria within the live cell fraction [14]. Necroptosis occurs over a period of time and the high level of ROS being detected is due at some point to the mitochondria in live necroptotic cells being functional and in a hyper-polarized state leading to the generation of most of the ROS detected Fig. 1 [2, 14, 21–24]. Other intracellular sources of ROS have been shown to be less affected by blockade with Nec-1 (unlike mitochondrial generated ROS) indicating that a small but significant proportion of ROS is not generated by mitochondria [8]. Although this does not indicate an absolute mitochondria requirement in the necroptotic process [2, 14, 21–24].

The use of multi-parameter flow cytometry to analyse RCD and ACD processes showed that live necroptotic cells (indicated by a 37% up-regulation of RIP3 which was abrogated by Nec-1) had functioning mitochondria with high levels of MMP and ROS which can be divided into the basic necroptotic phenotype which were negative for both γH2AX and cleaved PARP, while a high proportion of the necroptotic population displayed DNA Damage which was not increased by the high levels of ROS in these cells as may have been expected, see pathway of ROS induction of DNA Damage Fig. 1 [8, 14, 19]. The shikonin induced necroptosis within the live cell fraction also generated at a low incidence two more definable necroptotic populations which displayed cleaved PARP and parthanatos respectively, see pathway in Fig. 1 [8, 14, 19]. Early, late and RIP1-dependent apoptotic cells had little mitochondrial function but such early and live RIP1-dependent apoptotic cells showed increased ROS compared to untreated cells which was abrogated by zVAD. Early apoptotic and live RIP1-dependent apoptotic cells showed increased cleaved PARP (reduced by zVAD), with DNA Damage being reduced by Nec-1 blockade of shikonin (Fig. 1). zVAD as expected reduced levels of cleaved PARP and γH2AX hyper-activation of PARP in the dead apoptotic populations but increased the level of DNA Damage in dead RIP1-dependent apoptosis which Nec-1 reduced.

Once mitochondria became dysfunctional the ROS generated must have fallen relatively rapidly given that ROS levels in the live cell population without mitochondrial function have similar lower levels of ROS as those cells undergoing RIP1-dependent, early and late apoptosis. It would have been interesting to show how the level of ROS generated specifically from increasingly hyper-polarized mitochondria changed over time during the process of necroptosis and ultimately cell death. This time-course approach could also be used to monitor the effect Nec-1 blockade has on shikonin induced ROS and changes in MMP as it has been shown to significantly reduce ROS generated by mitochondria but not cellular ROS [8]. When these live necroptotic cells lose plasma membrane integrity or undergo cell death, the mitochondria in a proportion of these cells along with the live DN phenotype become more hyper-polarized (by 70%), presumably these cells make more ATP by such activity while at the same time double the level of ROS generated. Such implied increased energy levels indicate that these ‘dead’ necroptotic cells could perhaps have the capacity to influence ongoing biological processes even when the cell has lost plasma membrane integrity or has undergone the classic definition of cell death. It would also be interesting to observe how these biologically hyper-active cells initially develop, mature and degrade by studying these cells over time which may give an insight into their potential to alter the outcome of RCD and ACD processes.

More of these dead necroptotic cells have also then switched from mainly a DNA Damage phenotype to a parthanatos phenotype compared to live necroptotic cells. This indicated that the high level of ROS generated by these cells resulted in the γH2AX hyper-activation of cleaved PARP to generate this parthanatos population of dead necroptotic cells, see pathway of ROS driving development of parthanatos in Fig. 1. While, dead oncotic DN cells did not undergo such a change in RCD phenotypes significantly even though these cells also generated high levels of ROS, see Fig. 1.

Inhibitor blockade of shikonin did not change the degree of MMP and ROS generation in the live necroptotic cell phenotype but did result in increased cleaved PARP with reductions in DNA Damage by drug and Nec-1 but not zVAD. This indicated an enhancement of DNA repair mechanisms after Nec-1 blockade of shikonin which resulted in no change in the low incidence of necroptotic cells which displayed parthanatos, see pathway in Fig. 1. Upon cell death, dead necroptotic cells after inhibitor treatment showed no change in levels of cleaved PARP compared to drug induced levels, but there were reductions in DNA Damage by Nec-1 blockade of shikonin this apparently leading to increased levels of dead necroptotic cells which displayed parthanatos (compared to live necroptotic cells), this being decreased by zVAD but unchanged by Nec-1. In contrast dead oncotic DN cells showed no significant change in the incidence of parthanatos and DNA Damage after inhibitor treatments, although Nec-1 did increase the incidence of cleaved PARP, zVAD correspondingly decreased cleaved PARP. This indicated that shikonin acted upon a proportion of live cells which resulted in a down-regulation of RIP3 (rather than up-regulation to induce necroptosis) making them the live DN phenotype, they also showed low levels of cleaved PARP and γH2AX but high levels of MMP and ROS. This phenotypic profile did not change significantly upon cell death even though a high proportion of these cells showed hyper-polarized mitochondria with high levels of ROS but did not result in any increase in cell death markers. Although inhibition of shikonin reduced the incidence of this population, Nec-1 reduced MMP and increased the cleaved PARP dead oncotic DN population. Why shikonin induced this differential effect upon a cell line such as Jurkat T cells is difficult to ascertain but perhaps indicates that cells in a culture respond differently to the drug due to perhaps nutrient levels, functionality of organelles such as mitochondria, intracellular ROS levels and also the stage of cell division. This leads to shikonin apparently inducing more apoptosis and less detectable necroptosis with a high proportion of live cells moving to a DN phenotype which showed little sign of an RCD process analysed for in this study.

This flow cytometric based study of some of the biological functions of cells undergoing necroptosis along with the dynamic changes in the incidence of parthanatos and DNA Damage in response to shikonin and blockade of both necroptosis or apoptosis highlights the interconnectivity of numerous RCD processes. Given that necroptotic cells can also undergo parthanatos, DNA Damage or neither, perhaps these processes should no longer be classed as completely separate forms of cell death, as cells appear to move from displaying several specific forms of RCD to others during their response to undergoing cell death. The definition of cell death may also be required to be reviewed given that lack of plasma membrane integrity does not necessarily mean that the cell at least initially after loss of plasma membrane integrity is not biologically functional given the presence of hyper-polarized functional mitochondria.

## Electronic supplementary material

Below is the link to the electronic supplementary material.

Supplementary file1 (JPG 77 kb)

Supplementary file2 (JPG 159 kb)

Supplementary file3 (JPG 91 kb)

## Data Availability

All data is available on request.

## Abbreviations

ACD: Accidental cell death
ATP: Adenosine triphosphate
CamK II: Ca2 + /Calmodulin-dependent kinase II
Cleaved PARP: Poly (ADP-ribose) polymerase 1
DDR: DNA damage response
DN: Double negative
DNA DSBs: double strand DNA breaks
Drp1: Dynamin guanosine tri-phosphatase
EAPO: Early apoptosis
γHA2X: Histone A2 family X
LAPO: Late apoptosis
MFI: Median fluorescence intensity
MLKL: Pseudokinase mixed lineage kinase domain-like protein
MMP: Mitochondrial membrane potential
MPT: Mitochondrial permeability transition pore complex
NAD +: Nicotinamide adenine dinucleotide
Nec-1: Necrostatin-1
PDC: Pyruvate dehydrogenase complex
PGAM5: PGAM Family Member 5, Mitochondrial Serine/Threonine Protein Phosphatase
QN: Quadruple negative
RCD: Regulated cell death
RIP1: Receptor-interacting serine/threonine-protein kinase 1
RIP1 APO: RIP1-dependent apoptosis
RIP3: Receptor-interacting serine/threonine-protein kinase 3
ROS: Reactive oxygen species
RT: Room temperature
TNFα: Tumour Necrosis Factor alpha
TRADD: TNF receptor type 1-associated with death domain protein
TRAF-2: TNFR-associated factor 2 forming complex I
zVAD: Carbobenzoxy-valyl-alanyl-aspartyl-[O-methyl]- fluoromethylketone
