# Supplementary figures and images for: Flow cytometric detection of hyper-polarized mitochondria in regulated and accidental cell death processes
Source: Apoptosis. 2020 Jun 3;25(7):548–57. doi: 10.1007/s10495-020-01613-5 (PMC7347690; doi:10.1007/s10495-020-01613-5)

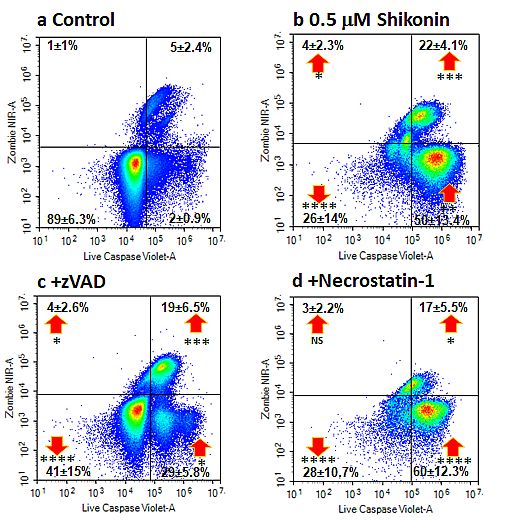

Supplement: Supplementary file 1 — Supplementary file1 (JPG 77 kb) [file 10495_2020_1613_MOESM1_ESM.jpg]

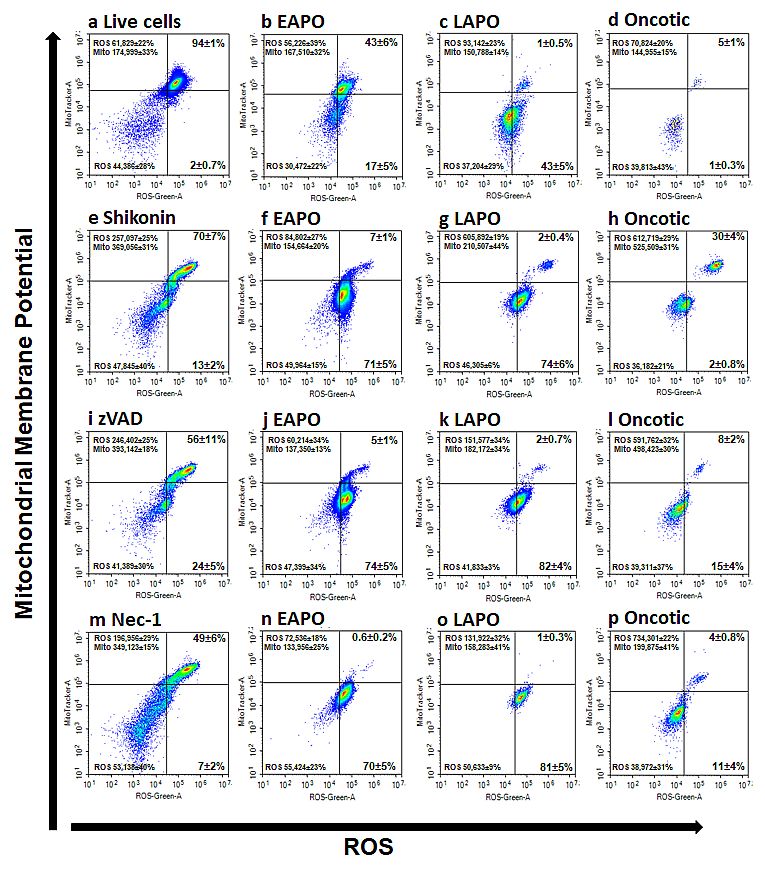

Supplement: Supplementary file 2 — Supplementary file2 (JPG 159 kb) [file 10495_2020_1613_MOESM2_ESM.jpg]

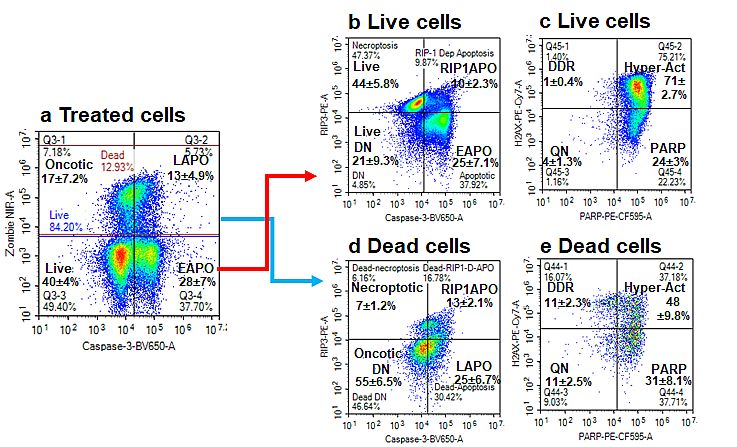

Supplement: Supplementary file 3 — Supplementary file3 (JPG 91 kb) [file 10495_2020_1613_MOESM3_ESM.jpg]
